# Good Way to Cook Apples

**Published:** 1875-12

**Authors:** 


### GOOD WAY TO COOK APPLES.

In the hurry of housework, the baking of apples, considered a small affair, is over-looked, and if the fruit can be sufficiently reduced by the heat, it matters not how it is done. This will make anything but an acceptable dish—acceptable to the eye as well as to the palate. You want every apple to remain perfect, retaining its shape, and not to be an undistinguishable mass of pulp, core and peel, baked next to dryness, or insufficiently cooked at the centre of the fruit.

To bake an apple properly is a nice thing, requiring attention. The first thing is uniformity of heat. I emphasize this as it is indispensable. Too much or too little heat is at all times faulty. Hence a coal stove is the place to bake an apple. You must acquire by experience the amount of heat needed. The time depends somewhat on the thickness of the peel, and the amount of moisture held by it. It is this holding the moisture while baking, that is the secret of successfully baking an apple. It is confining the steam which gets up a commotion and reduces the pulp to a fine texture, a thorough reduction, leaving the skin a thin silken covering, holding the flavor and aroma. This is an advantage to a highly flavored fruit, but it also improves an apple of inferior quality, lessening the acid.

To secure all this, an apple should be exposed to gentle heat from three to five hours, according to size, and whether sweet or sour; a sweet apple requiring more time. As soon as removed from the oven lay open the fruit, remove the core, sprinkle fine white sugar over it, close up and set away till cool. The Spitzenburgh is the king of fruit for this purpose. The point to be observed in baking is to see that the skin is not broken by the heat as it generates the steam.

